# Arterial Destiffening Starts Early after Renal Artery Denervation

**DOI:** 10.1155/2019/3845690

**Published:** 2019-03-03

**Authors:** Andrius Berukstis, Rokas Navickas, Gintarė Neverauskaite-Piliponiene, Ligita Ryliskyte, Jonas Misiura, Donatas Vajauskas, Nerijus Misonis, Aleksandras Laucevicius

**Affiliations:** ^1^Clinic of Heart and Vessel Diseases, Institute of Clinical Medicine Faculty of Medicine, Vilnius University, 08406 Vilnius, Lithuania; ^2^Faculty of Medicine, Vilnius University, 03101 Vilnius, Lithuania; ^3^Vilnius University Hospital Santaros klinikos, Radiology and Nuclear Medicine Center, 08406 Vilnius, Lithuania

## Abstract

**Introduction:**

Renal artery denervation (RDN) is a new widely discussed method in treatment of hypertension. Most of the RDN studies assessed BP and arterial changes 3 and 6 months after the procedure, but there is a lack of trials that investigated early changes after RDN.

**Aim:**

To investigate aortic stiffness 24-48 hours after the procedure and thus to examine whether RDN might have an early additive value for a cardiovascular risk decline beyond the lowering of blood pressure.

**Methods:**

RDN was performed for 73 patients with resistant hypertension. Arterial stiffness and central haemodynamics were measured before the procedure, the next day after the procedure, and subsequently after 1, 3, 6, and 12 months.

**Results:**

Within 48 hours, RDN significantly reduced aortic pulse wave velocity (AoPWV) from 11.3±2.7 to 10.3±2.6 m/s (*p*=0.001); reduction was sustained at months 1, 3, 6, and 12. Early changes in the AoPWV value did not correlate with changes in office systolic or diastolic BP (*p*=0.45;* p*=0.33). Furthermore, the higher the initial AoPWV value, the greater the reduction of AoPWV observed after 6 months: Q_1_ 8.4±1, Δ0.05±1.6 / Q_2_ 10.1±0.4, Δ1.1±1.4 / Q_3_ 12.2±0.8, Δ1.8±1.7 / Q_4_ 15.3±1.7, Δ2.8±2.1 (*p*=0.002).

**Conclusions:**

Early and sustained effects on AoPWV observed in our study suggest that RDN may have additional effects on reducing arterial stiffness and cardiovascular risk.

## 1. Introduction

Arterial hypertension is one of the most common diseases worldwide. According to the PURE study, overall 40.8% of the adult population in 2013 had hypertension [1], reaching as high as 57.6% in some age groups [2, 3]. The prevalence of resistant hypertension is increasing progressively with growing numbers of cardiovascular risk factors within the population [2]. Presence of risk factors concomitant to arterial hypertension lowers the success rate of hypertension treatment [4]. A meta-analysis of large studies indicates 12-15% prevalence of resistant hypertension among patients being treated for hypertension [5].

Resistant hypertension (RH) is recognised as a clinical phenotype carrying a high cardiovascular risk [6]. A retrospective cohort study by Daugherty et al. showed that 3960 patients with RH, followed for an average period of 48 months, had a higher incidence rate of cardiovascular events when compared to nonresistant hypertension patients (18.0% vs. 13.5%) [7]. Hypertension is defined as RH when the recommended treatment strategy fails to lower office systolic and diastolic blood pressure values to <140 mmHg and/or <90 mmHg, respectively, and the inadequate control of BP is confirmed by ambulatory blood pressure monitoring in patients whose adherence to therapy has been confirmed [8].

Sympathetic nervous system modification, by renal artery denervation (RDN), is a new interventional method in treatment of hypertension. Over the past decade there have been several studies with diverse conclusions on RDN effectiveness. The Symplicity HTN-1 and HTN-2 Trials showed significant blood pressure lowering effect [9, 10]. Unfortunately, Symplicity HTN-3 failed to prove a significant decrease in peripheral blood pressure six months after RDN when compared to placebo [11]. In 2017, SPYRAL OFF and ON studies confirmed the blood pressure lowering effect of RDN compared to placebo [12, 13]. The diversity of the results remains unclear.

By highlighting the relationship between RDN and arterial stiffness as well as central haemodynamics [14], Brandt MC et al. sparked new scientific discussions about a potential additive value of RDN apart from blood pressure reduction.

Aortic PWV has been assumed as the “gold standard” for measuring aortic stiffness [15] as an independent value for predicting cardiovascular events due to its responsibility for the majority of pathophysiological phenomena that end up causing cardiovascular outcomes [16]. It is recognised that aortic stiffness has an independent predictive value for all-cause mortality [17] and has an independent predictive value for fatal and nonfatal cardiovascular disease (CVD) outcomes in hypertensive patients [18].

The Brandt MC et al. study for the first time revealed aortic stiffness improvement 1 month after the procedure [14]. In addition, the study also tracked aortic PWV changes after longer periods of time. As known from a study by Seravalle et al., the effect of RDN procedure can be expected over a longer time period, which is thought to be related to gradually decreasing sympathetic nerve activity [19]. Up to date, it is not known when RDN effect starts and whether renal nerve ablation causes an early decrease in sympathetic nervous activity or the effect is delayed. As there is a lack of trials that investigated early changes after RDN, we aimed to investigate aortic stiffness and central haemodynamics 24-48 hours after the procedure. To confirm the durability of RDN, we had a follow-up of 1 year.

## 2. Materials and Methods

The study was authorized by the Regional Biomedical Research Ethics Committee (No. 158200-13-641-205). Prior to formal enrollment in the study, all interested patients signed an informed consent form.

Between March 2012 and December 2017, 243 patients (>18 years of age) with suspected RH were referred to a hypertension specialist. They underwent a detail examination using local protocol to rule out secondary hypertension [20], which included magnetic resonance tomography (MRT) of aorta, renal arteries, and adrenal glands. In case of contraindications to MRT, computed tomography was performed. Blood tests included aldosterone, renin (aldosterone to renin ratio calculated), metanephrine, and normetanephrine. Patients with secondary causes of hypertension were excluded from the study and were referred to other specialists. Simultaneously, during the first visit, patient's hypertension treatment was checked and changes were made, if needed. One month after the treatment correction, office and 24-hour ambulatory blood pressure measurements were performed. Those who did not respond to medical treatment after one month (did not reach recommended target office blood pressure and/or recommended ambulatory blood pressure, despite being treated with at least three antihypertensive drugs including diuretic) were included into the study. Patients were included in the study if they had renal artery anatomy eligible for treatment (main renal arteries >4 mm in diameter). Patients with myocardial infarction, unstable angina pectoris, cerebrovascular accident within the last 6 months, or haemodynamically significant valvular disease were excluded from the study. After excluding secondary hypertension and confirming treatment resistant hypertension, there were only 81 patients left: 8 patients refused the intervention and 73 proceeded to RDN.

### 2.1. Renal Artery Sympathetic Denervation

Renal denervation was performed using Symplicity Flex or Symplicity Spyral (Medtronic) catheter and corresponding radio-frequency generator. Arterial access catheter was inserted after puncturing the common femoral artery using modified Seldinger technique. After the cannulation of the artery, 5000 heparin units were administered to manage anticoagulation. A guiding catheter was then advanced over 0.035-inch wire to cannulate renal artery and an angiography of the renal arteries was performed with manual contrast-media injection. An RDN catheter was then positioned distally in the renal artery. Radiofrequency ablation was initiated if the contact with the arterial wall was proper: a flat impedance curve was displayed on the generator. Then catheter was pulled back proximally towards the aorta and rotated about 90 degrees and radiofrequency ablation was repeated. The number of radiofrequency ablations depended on the vascular anatomy. The complete procedure was then repeated with the other renal artery. If angiography showed accessory renal arteries of >3 mm in diameter, the radiofrequency ablation procedure was repeated in these arteries following the same methodology. After the procedure, all patients received Aspirin or Clopidogrel for at least 1 month.

### 2.2. Arterial Stiffness and Wave Reflection Measurements

The parameters of arterial stiffness and wave reflection were estimated by applanation tonometry (SphygmoCor v.8.0; AtCor Medical, Sydney, Australia). A high-fidelity micromanometer (Millar R; Millar Instruments, Houston, TX, USA) was placed on the skin surface to consequently obtain high quality radial, carotid, and femoral waveforms synchronized with ECG R-wave. The distance between the arterial sites was measured manually using a tape measure. Carotid-femoral pulse wave velocity, hereinafter referred to as aortic PWV (AoPWV), was calculated automatically as the distance divided by time (meters per second) and multiplied by 0.8 according to European expert consensus [21]. The aortic pressure waveform with calculation of the heart rate-adjusted aortic augmentation index (AIx) was automatically derived from a radial pressure waveform using a previously validated transfer function.

### 2.3. Follow-Up

Arterial stiffness and central haemodynamics were measured before the procedure, the next day after the procedure (between 24 and 48 hours), and subsequently 1, 3, 6, and 12 months after the procedure. In addition, office and 24-hour ambulatory blood pressure measurements were performed at 1, 3, 6, and 12 months after the procedure. During each visit, compliance with medical treatment was checked using patient's prescription passport. Our aim was to maintain stable medical treatment during the entire study period. At the time of examination, all patients had a stable cardiac status; there were no significant changes in medications from baseline up to 12 months.

### 2.4. Statistical Analysis

All analyses were performed using SPSS 20.0. Data were presented as mean ± SD or as proportions. The paired t-test (parametrical) or Wilcoxon signed-rank test (nonparametrical) was used to compare values between time points. The Pearson correlation or Spearman's correlation was used to identify correlations between PWV and MAP changes during follow-up. To identify whether the initial AoPWV value had influence on an AoPWV value after 6 months, the initial AoPWV was divided into the first, second, third, and fourth quartile. Then ANOVA was used to determine whether AoPWV change differs significantly between the quartiles. The results were considered significant when* p *value was <0.05.

## 3. Results

The study included 73 patients with resistant arterial hypertension who underwent bilateral RDN. There were 39 female and 34 male patients with mean age of 56.02 ± 7.7 (37-72) years. Obesity was prevalent in the group with an average body mass index of 34.29 ± 5.6. Left ventricular hypertrophy was a frequent finding (80%, n=48) as well as family history of hypertension (64.6%, n=42). Prevalence of diabetes was 39.7% (n=29) and prevalence of coronary heart disease was 22.5% (n=16).

Average office blood pressure in the study group prior to any interventions was 190/107 (±22/ 13) mmHg, in patients treated with an average of 6.2 (±1.5) antihypertensive drugs at maximal or maximal tolerated doses. There were no significant changes between the numbers of drugs taken 3, 6, and 12 months after the procedure, respectively, 5.65 (±1.37),* p*=0.568, 5.72 (±1.37),* p*=0.176. Baseline characteristics of the patients are shown in Table 1.

Comparing the pre- and postprocedure findings, 24-48 hours after the procedure, there was a significant reduction in aortic mean arterial pressure (MAP) from 128±19 to 116±16 mmHg (*p*<=0.001), aortic pulse pressure from 64±17 to 56±17 mmHg (*p*<=0.001), and augmentation index HR75 from 27 ± 10 to 23 ± 11% (*p*=0.001). These measurements remained reduced after 1, 3, 6, and 12 months.

Within 48 hours RDN significantly reduced AoPWV from 11.3±2.7 to 10.3±2.6 m/s (*p*=0.001) and carotid-radial pulse wave velocity from 9.9±1.9 to 9.3±14 m/s (*p*=0.03). Correlation analysis showed the MAP-independent improvement of AoPWV during 48 hours after RSD (r=0.165), suggesting AoPWV reduction being not limited to the blood pressure response. All recorded data on central haemodynamics and AoPWV changes are shown in Table 2.

**Figure 1:**
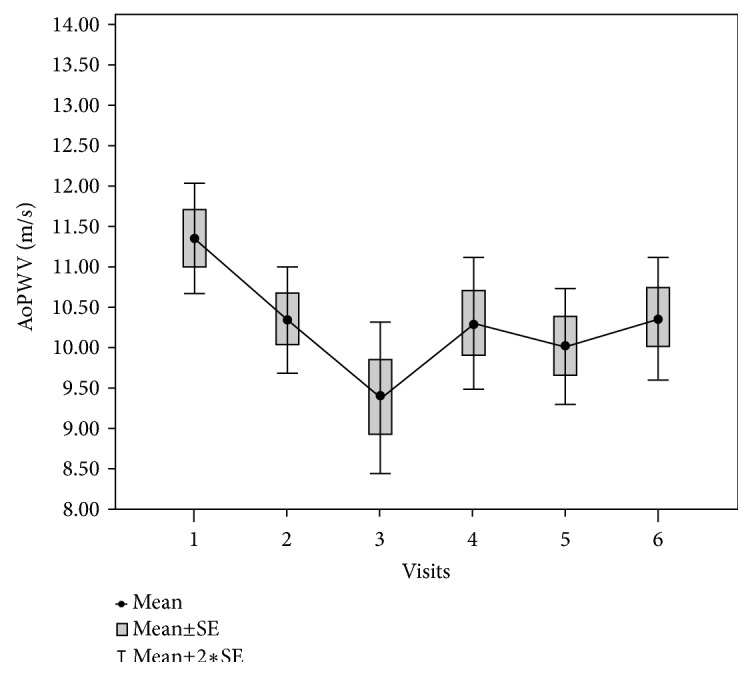
Aortic PWV changes. Visit 1: before RDN AoPWV 11.3 ± 2,7 m/s. Visit 2: within 24–48 hours after RDN AoPWV 10.3 ± 2,6 m/s (*p*<0.001). Visit 3: after 1 month AoPWV 9.4 ± 2,2 m/s (*p*<0.001). Visit 4: after 3 months AoPWV 10.3 ± 2,9 m/s (*p*=0.013). Visit 5: after 6 months AoPWV 10 ± 2,6 m/s (*p*<0.001). Visit 6: after 1 year AoPWV 10.4 ± 2.7,* p=*0.013.

When analysing the data of the follow-up, we found a significant drop in office blood pressure, seen at month 1, from baseline 190/107 ± 22/13 to 160/95 ± 23/14 mm Hg, which was sustained at months 3, 6, and 12. A significant drop in 24-hour ambulatory blood pressure was observed at month 3 from baseline 163/97 ± 18/14 to 151/89 ± 22/12 mm Hg and was sustained at months 6 and 12. Office and 24-hour ambulatory BP details with regard to other timeframes are presented in Table 2. The significant decrease in AoPWV was also sustained at months 1, 3, 6, and 12, respectively, by 9.4±2.2 (*p*<0.001), 10.3±2.9 (*p*=0.013), 10±2.6 (*p*<0.001), and 10.4±2.7 (*p*=0.013) m/s, as shown in Figure 1. Furthermore, the higher the initial AoPWV value, the greater the reduction of AoPWV value observed after 6 months: Q_1_ 8.4±1, Δ 0.05±1.6 / Q_2_ 10.1±0.4, Δ 1.1±1.4 / Q_3_ 12.2±0.8, Δ 1.8±1.7 / Q_4_ 15.3±1.7, Δ 2.8±2.1, (*p*=0.002). The quartile analysis for the relationship between the baseline AoPWV values and AoPWV changes at 6 months after the procedure is presented in Table 3. The changes of the AoPWV value did not correlate with office systolic or diastolic BP (*p*=0.45;* p*=0.33).

## 4. Discussion

The rationale of RDN is that ablation destroys afferent and efferent renal sympathetic nerves. Efferent innervation plays a major role in regulation of renal blood flow, glomerular filtration rate, sodium reabsorption, and subsequent water retention [22]. The model of interactions between the renin–angiotensin–aldosterone system and sympathetic renal innervations is complex. It is known that increased renal sympathetic nerve activation causes increases in renin release, thus increasing renal vascular resistance and sodium reabsorption [23]. In addition, afferent signals from renal mechanoreceptors and chemoreceptors elicit activation of the sympathetic nervous system leading to increased systemic vascular resistance. This data is mostly based on animal studies [24, 25]. Furthermore, increased sympathetic nervous activity and high blood pressure cause arterial stiffening and increase AoPWV [26, 27].

Our positive results on arterial stiffness and central haemodynamics after RDN at months 1, 3, and 6 coincide with those found by Brandt MC et al. [14]. These results suggest that aortic stiffness reduced by RDN provides additional benefit apart from blood pressure reduction by reducing CVD risk and mortality. Moreover, our follow-up results at month 12 show a sustainable effect of RDN on reducing arterial stiffness and central haemodynamics. This supports the results of a similar study by Ott et al. [28] and allows us to conclude that longer positive effects on arterial stiffness elicit a greater CVD risk and mortality reduction. A sham-controlled study by Peters et al. [29] has also arrived at similar positive results within the RDN group, but no statistical significance was reached in comparison with the sham group, probably due to a small sample size (SHAM-27 vs. RDN-26).

It is also important to mention that the reduction of PWV and central haemodynamics was seen in a study group that consisted of patients with high cardiovascular risk, obesity, and a lot of comorbidities that influenced their PWV to be high at baseline. To add more, our results suggest that patients with high cardiovascular risk might benefit the most; the greater the arterial stiffness before the procedure, the greater the decrease we can expect after RDN. Correlation between baseline AoPWV values and AoPWV reduction by RDN was also observed by Brandt MC et al. [14].

The reduction in AoPWV following RDN observed 24-48 hours after the intervention is new data that shows early cardiovascular benefit from RDN. AoPWV measurements have a great scientific interest, although clinical application is narrow. Our results suggest that it might serve as an early marker of decreased sympathetic innervation. It is known that arterial stiffness is associated with sympathetic innervation. Patients who underwent ipsilateral anaesthesia of the brachial plexus before the surgery for Dupuytren's contracture had a greater decrease in arterial stiffness of the radial artery of the disabled hand, whilst distensibility of the contralateral artery remained unaffected [30]. The same effect was seen for femoral artery distensibility following both ipsilateral subarachnoid anaesthesia in healthy subjects and ipsilateral sympathetic ganglionectomy in patients with peripheral artery disease [31]. This was earlier observed in animal studies [32]. We also found that early changes in AoPWV velocity were independent of blood pressure response. Therefore, we think that significant arterial destiffening might occur due to modification of sympathetic tone after RDN.

Assessment methods for sympathetic tone modification after renal artery ablation described in previously published studies include renal norepinephrine spillover measurements, microneurography, and peri-procedural high-frequency stimulation. One month after RDN, renal norepinephrine spillover decreased by 47% in a small cohort of the Symplicity HTN-1 trial [33]. Muscle sympathetic nerve activity, assessed by microneurography, decreased toward normal levels at 30 days after RDN [34]. Although these methods provide detailed information about sympathetic tone, their use in everyday clinical practice is limited because of their complexity and the need for intervention as in case of norepinephrine spillover measurement. To confirm successful renal denervation, Pokushov et al. used high-frequency stimulation before the initial and after each RF delivery within the renal artery. Rectangular electrical stimuli were delivered at the ostium of the targeted renal artery at a frequency of 20 Hz, with an amplitude of 15 V and pulse duration of 10 ms for 10 s. Renal sympathetic denervation was considered to have been achieved when the sudden increase of blood pressure (>15mm Hg from invasive arterial monitoring) was eliminated in the presence of high-frequency stimulation [35]. Since there is not sufficient data about safety and efficacy of the use of cardiac catheters in renal arteries, this method is unsuitable for everyday clinical practice.

## 5. Conclusions

Early and sustained effects on AoPWV observed in our study as early as within 24-48 hours after the procedure for up to 12 months are indicative of an additional RDN effect on reducing arterial stiffness and cardiovascular risk. We also observed a significant blood pressure reduction that sustained for up to 12 months after the procedure.

We think that AoPWV could possibly be considered a quality indicator for successfully performed renal artery ablation, with no easily applicable realistic alternatives existing in clinical practice. However, further blinded studies would be needed to confirm the findings.

## 6. Limitations

The biggest limitation is the absence of a control group, which consequently makes it not a comparison study, but a cohort study that reports a course of arterial stiffness and central haemodynamics after RDN. Second limitation is unavailability of direct measurement of sympathetic nervous activity by standard methods (microneurography or norepinephrine spillover) because of their complexity and the need for intervention.

## Figures and Tables

**Table 1:** Baseline patient characteristics.

Baseline patient characteristics	

Group size (n)	73

Age, years	56.02 (± 7.7)

Gender:	

Male, n (%)	34 (46.6%)

Female, n (%)	39 (53.4%)

BMI (kg/m^2^)	34.29 (± 5.6)

Left ventricular hypertrophy (echocardiography)	48 (80%)

Family history of hypertension	42 (64.6%)

Type 2 diabetes, n (%)	29 (39.7%)

Coronary artery disease, n (%)	16 (22.5%)

Number of antihypertensive drugs before the procedure	6.2 (± 1.5)

Office systolic BP (mm Hg)	190(± 22)

Office diastolic BP (mm Hg)	107(± 13)

Office heart rate (beats/min)	72 (±9)

Number of ablation points performed	16.21 (± 8.95)

**Table 2:** Blood pressure, central haemodynamics and arterial stiffness.

	Baseline		Month 1	Month 3	Month 6	Year 1
24-hour ambulatory BP						

Mean systolic BP (mm Hg)	163 ± 18		152 ± 18 (n-21, *p*=0.009)	151 ± 22 (n-54, *p*<0.001)	149 ± 20 (n-53, *p*<0.001)	152± 17(n-49, *p<*0.001)
Mean diastolic BP (mm Hg)	97 ± 14		93 ± 14 (n-21, *p*=0.28)	89 ± 12 (n-54, *p*<0.001)	90 ± 10 (n-53, *p*<0.001)	91± 10 (n-49, *p=*0.001)
Mean HR (beats/min)	71 ± 10		70 ± 8 (n-21, *p*=0.186)	70 ± 9 (n-52, *p*=0.154))	70 ± 9 (n-51, *p*=0.201)	68± 8 (n-48, *p*=0.242)
Daytime mean systolic BP (mm Hg)	168 ± 18		156 ± 18 (n-21, *p*=0.079)	153 ± 23 (n-54, *p*<0.001))	152 ± 20 (n-52, *p*<0.001)	155 ± 18 (n-48, *p*<0.001)
Daytime mean diastolic BP (mm Hg)	101 ± 15		97 ± 14 (n-21, *p*=0.284)	92 ± 13 (n-54, *p*<0.001)	94 ± 11 (n-52, *p*<0.001)	95 ± 11 (n-48, *p=*0.001)
Nighttime mean systolic BP (mm Hg)	155 ± 21		140 ± 20 (n-21, *p*=0.022)	144 ± 25 (n-54, *p*<0.001)	142 ± 22 (n-52, *p*<0.001)	144 ± 20 (n-48, *p*<0.001)
Nighttime mean diastolic BP (mm Hg)	90 ± 14		82 ± 16 (n-21, *p*=0.045)	83 ± 13 (n-54, *p*<0.001)	83 ± 10 (n-52, *p*<0.001)	83 ± 11 (n-48, *p*=0.102)

Office BP						

Systolic BP (mm Hg)	190 ± 22		160 ± 23 (n-17, *p*<0.001)	160 ± 21 (n-59, *p*<0.001)	156 ±25 (n-62, *p*<0.001)	162 ± 26(n-55, *p*<0.001)
Diastolic BP (mm Hg)	107 ± 13		95 ± 14 (n-17, *p*<0.001)	91 ± 13 (n-59, *p*<0.001)	90 ± 12 (n-62, *p*<0.001)	94 ± 17 (n-55, *p*<0.001)
Office HR	72 ±9		69 ± 12 (n-17, *p*=0.715)	66 ± 10 (n-37, *p*=0.002)	68 ± 11 (n-47, *p*=0.068)	69 ± 12 (n-48, *p=*0.185)

	Baseline	24-48 hours after the procedure	Month 1	Month 3	Month 6	Year 1

Central haemodynamics and arterial stiffness parameters						

Aortic pressure	65 ±17	56±17 (n-58, *p*<0.001)	54±16 (n-22, *p<*0.001)	58±17 (n-45, *p*=0.001)	56±19 (n-50, *p<*0.001)	57±15 (n-45, *p<*0.001)
Augmentation index HR	27 ±10	23±11 (n-57, *p=*0.001)	21±12 (n-22, *p=*0.029)	24±10 (n-44, *p=*0.01)	25±10, (n-48, *p=*0.358)	25±10 (n-46, *p=*0.068)
MAP	128 ± 19	116 ±16 (n-58, *p<*0.001)	117±18 (n-22, *p=*0.001)	118±16 (n-45, *p<*0.001)	117±23 (n-50, *p=*0.001)	119±19 (n-46, *p<*0.001)
AoPWV	11.3 ±2.7	10.3 ±2.6 (n-58, *p=*0.001)	9.4 ±2.2 (n-22, *p<*0.001)	10.3 ±2.9 (n-44, *p=*0.013)	10 ±2.6 (n-48, *p<*0.001)	10.4 ±2.7 n-45, *p=*0.013

BP: blood pressure; HR: heart rate; MAP: mean arterial pressure; AoPWV: aortic pulse wave velocity.

**Table 3:** Effects of the baseline pulse wave velocity on pulse wave velocity changes after 6 months.

	MV±SD	MV±SD	MV±SD	MV±SD
	Q1, <9.5 m/s	Q2, 9.5-10.699 m/s	Q3, 10.7-13.2 m/s	Q4, >13.2 m/s

AoPWV before procedure, m/s	8.4 ± 1	10.1 ± 0.4	12.2 ± 0.8	15.3 ± 1.7

AoPWV 6 months after, m/s	8.4 ± 1.7	8.9 ± 1.6	10.3 ± 2.1	13.2 ± 2.1

Reduction, Δ m/s	0.05 ± 1.6	1.1 ± 1.4	1.8 ± 1.7	2.8 ± 2.1

MV: mean value; SD: standard deviation; AoPWV: aortic pulse wave velocity.

## Data Availability

The data used to support the findings of this study are included within the article.
